# Multi-Dimensional Transcriptomics Reveals the Prominent Role of Neuroinflammation in Alzheimer’s Disease

**DOI:** 10.3390/ijms27115020

**Published:** 2026-06-02

**Authors:** Xingyu Wang, Zhouting Rong, Feng Xue

**Affiliations:** 1 Hwamei College of Life and Health Sciences, Zhejiang Wanli University, Ningbo 315100, China; nwxy6670@163.com; 2 Zhejiang Key Laboratory of Intelligent Food Logistic and Processing, Yuyao Innovation Institute, Zhejiang Wanli University, Ningbo 315400, China

**Keywords:** Alzheimer’s disease, neuroinflammation, single-cell RNA sequencing, spatial transcriptomics, microglia, astrocyte

## Abstract

Alzheimer’s Disease (AD), the most common form of dementia, is pathologically defined by extracellular beta-amyloid (Aβ) plaques and intraneuronal neurofibrillary tangles (NFTs), accompanied by chronic neuroinflammation. Recent advances in single-cell RNA sequencing (scRNA-seq/snRNA-seq) and spatial transcriptomics have provided unprecedented resolution for unraveling the cellular and molecular landscape of neuroinflammation in AD. While scRNA-seq enables high-throughput profiling of cellular heterogeneity across brain regions, spatial transcriptomics preserves tissue architecture to map cell-type-specific gene expression within its anatomical context. This review synthesizes the neuroinflammatory mechanisms of AD, outlines the technical evolution and comparative capabilities of single-cell and spatial omics platforms, including resolution, throughput, and compatibility with multiple sample types, and critically evaluates findings from studies in both animal models and human brain tissues. These approaches have revealed state-specific transitions in microglia and astrocytes, including shifts in transcriptional programs, metabolic reprogramming, and pro-inflammatory polarization across disease stages. Notably, spatial transcriptomic analyses demonstrate pronounced regional heterogeneity: periplaque microenvironments exhibit distinct immune-cell compositions and gene expression signatures. Collectively, these omics technologies are redefining the cellular basis of AD progression and hold the potential to impact the discovery of early diagnostic biomarkers and precision therapeutic targets.

## 1. Introduction

Alzheimer’s Disease (AD) is a progressive neurodegenerative disorder clinically characterized by a gradual decline in cognitive and memory functions [1]. It is estimated that over 55 million people worldwide are living with AD, a number projected to reach 78 million by 2030 [2]. Typical brain pathologies in AD include the deposition of Aβ plaques and neurofibrillary tangles in the cerebral cortex and hippocampus, accompanied by neuronal and synaptic loss [1,3]. Against the backdrop of global population aging, the rising prevalence of AD imposes a substantial economic and caregiving burden on global society [4]. Traditionally, the pathological mechanisms of AD have been dominated by the Aβ cascade hypothesis and tauopathy, yet these theories have limitations in explaining the full spectrum of the disease and guiding effective treatments [5]. Although monoclonal antibodies targeting Aβ have shown some progress in clinical trials recently, their efficacy remains limited and is associated with specific risks. Meanwhile, therapies targeting tau have yet to achieve decisive breakthroughs, prompting the scientific community to reflect deeply and actively explore new pathological perspectives [6].

Emerging evidence indicates that neuroinflammation plays a prominent role in AD pathogenesis. The brain’s resident immune cells, microglia and astrocytes, can recognize and clear Aβ deposits. However, upon aberrant activation, they secrete large amounts of pro-inflammatory cytokines and complement proteins, exacerbating neuronal damage [7,8]. Neuroinflammation in the central nervous system is primarily mediated by microglia and astrocytes, which in turn amplify AD pathology through multiple mechanisms [9]. First, neuroinflammation and Aβ pathology form a vicious cycle: microglial dysfunction impairs Aβ clearance, while inflammatory signals promote amyloid precursor protein (APP) processing to generate more Aβ. Furthermore, Aβ fibrils can activate intracellular inflammasomes, further intensifying the inflammatory response [10]. Second, neuroinflammation interacts with tau pathology: inflammatory factors can activate kinases, leading to tau hyperphosphorylation and promoting microglia-mediated prion-like spreading of tau [11]. Additionally, neuroinflammation directly contributes to synaptic dysfunction and neuronal death, for instance through complement pathway-mediated aberrant synaptic pruning and direct neurotoxicity of inflammatory factors [12].

For example, Aβ oligomers activate microglia, and through pathways involving NLRP3 inflammasome activation or TLR signaling these cells release inflammatory mediators such as IL-1α, TNF-α, and C1q. These factors subsequently induce astrocytes to transform into a neurotoxic A1 phenotype, producing neurotoxic substances that lead to neuronal death [8,13]. The A1/A2 dichotomy, while heuristically useful, has been criticized as overly simplistic, as transcriptomic evidence suggests that astrocytes exist along a continuous spectrum of activation states rather than as discrete binary categories. Reactive A1 astrocytes contribute to AD pathogenesis through multiple mechanisms: they upregulate complement component C3, which interacts with neuronal C3a receptors to induce synaptic and dendritic damage, and they express APP, BACE-1, and γ-secretase, thereby increasing Aβ production and exacerbating neuroinflammation [8].

Genetic evidence further supports the important role of neuroinflammation. Genome-wide association studies (GWASs) have identified AD risk genes such as *TREM2* and *CD33* that are primarily expressed in microglia and directly involved in inflammatory regulation [14]. Moreover, AD-associated genetic risk variants are predominantly expressed in immune cells, suggesting a close link between immune pathways and AD pathology [13]. Deficiency or mutation of TREM2 impairs the ability of microglia to phagocytose Aβ and enhances inflammatory responses, thereby aggravating plaque deposition and neuronal injury [13]. Recent studies have also revealed that besides glial cells, oligodendrocytes exhibit a ‘disease-associated oligodendrocyte’ (DAO) phenotype in AD, linked to abnormalities in myelination and Erk signaling pathways [3].

**Figure 1:**
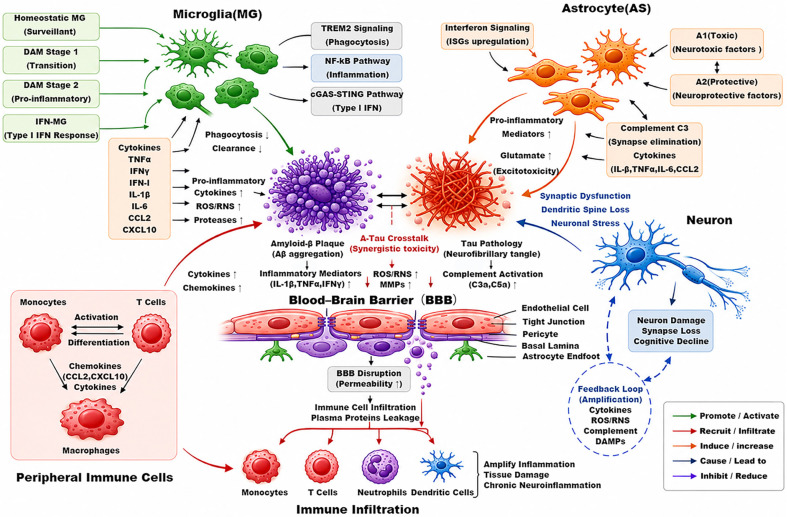
Neuroinflammatory network and cellular crosstalk in AD.

In summary, positioning neuroinflammation at the center of the AD pathological network provides an integrative framework for understanding disease mechanisms and developing novel anti-inflammatory therapies [15]. The pathogenesis of AD involves a complex network of immune responses triggered by Aβ and tau accumulation, encompassing pathways such as the NLRP3 inflammasome, the complement system, and neurotoxic glial reactions [8,13]. The recent widespread adoption of single-cell RNA sequencing and spatial transcriptomics has significantly advanced our mechanistic understanding of neuroinflammation in AD, revealing cell-type-specific dynamics and spatially resolved inflammatory signatures at unprecedented resolution (Figure 1). This review is primarily a disease-mechanism-focused review that integrates methodological considerations and translational perspectives. We aim to provide a comprehensive synthesis of how multi-dimensional transcriptomics has reshaped our understanding of neuroinflammation in AD, while also addressing the technical foundations, critical limitations, and clinical implications of these approaches.

Schematic overview of the major cellular and molecular interactions driving chronic neuroinflammation during AD progression. Activated microglia undergo phenotypic transitions from homeostatic states toward disease-associated and interferon-responsive subpopulations, accompanied by activation of inflammatory pathways, cytokine production, and impaired phagocytic clearance of Aβ. Reactive astrocytes contribute to inflammatory amplification through complement activation, cytokine release, excitotoxic signaling, and modulation of neuronal stress responses. Accumulation of Aβ plaques and tau pathology synergistically promotes oxidative stress, protease activation, and chronic inflammatory signaling, resulting in progressive synaptic dysfunction and neuronal degeneration. Blood–brain barrier disruption (BBB) further facilitates peripheral immune-cell infiltration, thereby exacerbating neuroimmune dysregulation and tissue damage. Bidirectional feedback interactions among glial cells, neurons, protein aggregates, and infiltrating immune cells establish a self-amplifying inflammatory loop that contributes to synapse loss, cognitive impairment, and neurodegeneration in AD.

## 2. Methodology Review

### 2.1. Single-Cell RNA Sequencing (scRNA-Seq/snRNA-Seq)

Single-cell transcriptomics technology, first developed in 2009, enables the capture of the entire transcriptome from individual intact cells [16]. While traditional bulk transcriptome sequencing requires inputs of hundreds of thousands of cells and consequently reflects only the population average, scRNA-seq overcomes these technical limitations by accurately quantifying gene expression levels at a single-cell resolution [17]. A core technical advantage that has emerged from this approach is its ability to systematically resolve cellular heterogeneity, thereby avoiding the signal averaging inherent in bulk sequencing methods [17]. Compared to traditional approaches, scRNA-seq not only identifies rare cell populations but also reveals continuous trajectories of cell state transitions through high-dimensional data analysis, thus enabling more accurate reflection of the dynamic changes in cell populations during disease progression [18]. In terms of the advantages of single-cell sequencing for large-scale data clustering, applying unsupervised clustering algorithms to thousands to millions of single-cell transcriptomes allows for the systematic categorization of cells, identifying previously undefined cell subtypes within tissues [19].

Currently, the most widely adopted mainstream method is the microfluidic platform from 10× Genomics, which encapsulates single cells or nuclei with barcoded beads within water-in-oil droplets. The mRNA from each cell is reverse-transcribed within the droplet and tagged with a unique barcode, enabling parallel sequencing of hundreds to hundreds of thousands of cells [16]. Compared to whole-cell sequencing, snRNA-seq can utilize frozen or fixed brain tissue to extract nuclei, offering better compatibility for brain cells like neurons that are difficult to dissociate [16]. The advantages of sc/snRNA-seq technologies include near-single-cell resolution and whole-genome expression profiling capability with high detection sensitivity. However, sample preparation requires tissue dissociation into single cells, resulting in the loss of original spatial information [20]. A major limitation highlighted in large-scale single-cell studies is the prevalence of batch effects, which requires the use of specialized bioinformatics algorithms for quality assessment and data integration [21].

### 2.2. Spatial Transcriptomics Technology

Spatial omics technologies sequence mRNA while preserving the tissue’s in situ architecture, establishing a direct correspondence between gene expression and spatial distribution. As a crucial complement to scRNA-seq, the advantage of spatial transcriptomics lies in its ability to map gene expression data directly onto spatial coordinates within the tissue structure, retaining the positional relationships of cells within their native microenvironment [22]. Representative platforms include sequencing-based methods (Visium, Slide-seq, Stereo-seq, etc.) and imaging-based methods (MERFISH, seqFISH+, etc.). In 2016, Ståhl et al. first reported spatial transcriptomics technology based on printed chips, where oligonucleotide arrays with spatial barcodes are placed on tissue sections to generate two-dimensional maps of gene expression within the tissue [22].

The commercialized 10× Genomics Visium system builds upon this principle, utilizing arrays of capture probes with spots approximately 55 μm in diameter (containing ~1–10 cells per spot, ~5000 spots/slide), enabling genome-wide sequencing of tissue sections [23]. Slide-seq utilizes arrays of beads approximately 10 μm in diameter to capture RNA, achieving near-single-cell spatial resolution [24]. Imaging-based in situ hybridization methods employ multiple rounds of fluorescence in situ hybridization (FISH) to capture RNA. MERFISH utilizes combinatorial barcoding with error-robust encoding schemes, enabling the localization of hundreds to thousands of genes at single-cell resolution [16,25]; seqFISH+ employs 60 ‘pseudo-colors’ across multiple hybridization rounds, reportedly enabling the detection of approximately 10,000 genes [16]. The characteristics of these methods are compared in Table 1. Recent advances in integrative multi-omics approaches have further enhanced the resolution and information content of spatial transcriptomics, enabling simultaneous analysis of gene expression and spatial location at the single-cell level [26].

**Figure 2:**
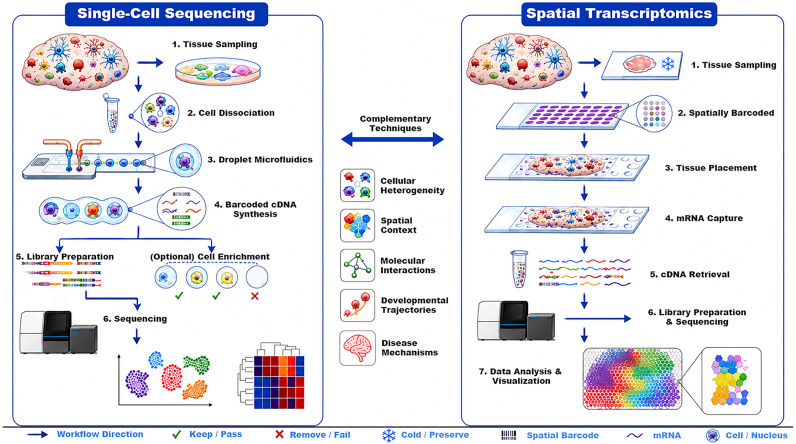
Integrated workflows of single-cell sequencing and spatial transcriptomics for decoding tissue heterogeneity and microenvironmental organization.

Therefore, sequencing-based spatial omics methods (e.g., Visium, Slide-seq, Stereo-seq) offer whole-transcriptome coverage but generally require fresh tissue and are limited by resolution constraints. In contrast, imaging-based methods (MERFISH, seqFISH+) can achieve subcellular resolution but currently rely on targeted gene panels, involve higher costs, and require longer imaging times. Each method has its own strengths and weaknesses; experimental design must balance factors such as spatial resolution, detection throughput, sample processing complexity, and budget [20]. Nevertheless, the combined use of single-cell sequencing and spatial omics has been proved an effective complementary technique in illustrating AD pathology (Figure 2).

Schematic illustration comparing the experimental and analytical pipelines of single-cell sequencing and spatial transcriptomics. In the single-cell sequencing workflow, tissues are dissociated into single-cell suspensions, followed by droplet-based microfluidic partitioning, barcode labeling, cDNA synthesis, library preparation, and high-throughput sequencing to resolve cellular heterogeneity and transcriptional states at single-cell resolution. In the spatial transcriptomics workflow, intact tissue sections are placed onto spatially barcoded slides, enabling spatially resolved mRNA capture, reverse transcription, sequencing, and reconstruction of gene-expression landscapes while preserving tissue architecture. Integrative analysis of these complementary technologies enables comprehensive characterization of cell populations, spatial niches, intercellular communication networks, developmental trajectories, and disease-associated microenvironments. Combined single-cell and spatial multi-omics approaches provide powerful frameworks for investigating complex pathological processes, including neuroinflammation, tumor ecosystems, and tissue remodeling in human diseases.

## 3. Major Findings

### 3.1. Animal Model Studies

Single-cell and spatial omics analyses using AD animal models (e.g., 5xFAD, 3xTg, APP/PS1, App NL-G-F) have revealed key cell subtypes and gene pathways involved in neuroinflammation. Regarding microglia, Keren-Shaul et al. (2017) [27] first identified a TREM2-dependent ‘disease-associated microglia’ (DAM) subpopulation in 5xFAD mice, characterized by enrichment of genes related to lipid metabolism and phagocytosis. Molecular features include upregulation of genes like *Apoe*, *Trem2*, *Cst7*, and downregulation of homeostatic markers such as *P2ry12* and *Tmem119* [27]. DAM activation is dependent on the TREM2 signaling pathway, which plays a critical regulatory role in AD pathology [28,29].

Regarding astrocytes, Habib et al. (2020) identified a novel ‘disease-associated astrocyte’ (DAA) population in the hippocampus of 5xFAD mice, which appears early and increases with disease progression [30]. In the mid-to-late stages of AD, as Aβ plaques deposit and tau pathology spreads, microglia in the brain become chronically activated, releasing large amounts of inflammatory factors (e.g., IL-1α, TNF, C1q). Although astrocytes are not professional immune cells, they can be indirectly activated by this inflammatory milieu, acquiring immune-regulatory functions and actively participating in the neuroinflammatory process [31]. Park et al. (2023) further reported novel DAO subtypes in App NL-G-F and 5xFAD mice, linking abnormal Erk signaling activation to impaired myelination [3].

Spatial omics studies have elucidated cellular interactions within the plaque environment: Mallach et al. (2024) used CosMx and Stereo-seq spatial transcriptomics to analyze the hippocampus and adjacent cortical regions of App^NL-G-F knock-in mice, finding that microglia heavily aggregate around plaques, disrupting astrocyte-to-microglia crosstalk and causing synaptic imbalance; they also noted that microglial responses were relatively consistent across different brain regions, whereas astrocyte responses exhibited regional heterogeneity [32]. A recent study based on Stereo-seq spatial transcriptomics further identified a reactive microglial subtype (Micro. 1) in the AD hippocampus, significantly enriched around Aβ plaques and highly expressing complement components (e.g., C3, C7) and DAM genes, suggesting a key role in neuroinflammation and synaptic pruning [33]. Key findings from relevant animal studies are summarized in Table 2.

While AD animal models have provided invaluable insights, several critical translational limitations must be acknowledged. First, common transgenic models (e.g., 5xFAD, APP/PS1) overexpress mutant human APP/PS1 transgenes, resulting in supraphysiological and early-onset Aβ pathology that does not fully recapitulate the sporadic, age-dependent nature of human AD. Second, these amyloid-driven models lack robust tau pathology and neurofibrillary tangles, limiting their utility for studying tau-related neuroinflammation. Third, young adult mice do not exhibit aging-related immune changes (e.g., immunosenescence, TIM phenotype) that profoundly impact microglial function in aged human brains. Fourth, vascular pathology and chronic, progressive neurodegeneration are less pronounced in most transgenic models compared to human AD. Fifth, species-specific differences in glial biology, such as distinct transcriptional programs and turnover rates between human and mouse microglia, may influence the translatability of findings. Knock-in models (e.g., App^NL-G-F), humanized mouse models, and iPSC-derived systems offer potential advantages for bridging this translational gap.

### 3.2. Human Studies

Significant progress has also been achieved in recent years in single-cell/single-nucleus and spatial omics studies of human AD brain tissue. Mathys et al. (2024) [1] constructed a single-cell transcriptomic atlas encompassing 1.3 million cells from 283 human brain samples across six brain regions. They identified up to 76 cell types and demonstrated that specific excitatory neuron populations were markedly reduced in AD. Their study also implicated the Reelin pathway in AD resilience and revealed specialized transcriptional programs in astrocytes associated with cognitive resistance [1]. Single-cell transcriptomic analysis of the AD brain reveals the existence of diverse neuroinflammation-related cellular subpopulations, including disease-associated microglia characterized by elevated expression of MHC-II genes and complement components, as well as reactive astrocyte subsets marked by upregulation of the AD risk gene *CLU* [19].

Sziraki et al. (2023) employed a novel combinatorial indexing technology (EasySci-RNA) to resolve cellular heterogeneity in mice (1.5M cells) and humans (118k cells), identifying numerous rare cell subtypes and AD-associated alterations [34]. Miyoshi et al. (2024) compared spatial and single-cell omics data from the brains of patients with sporadic AD and Down syndrome-associated AD (DSAD), revealing aberrant glial inflammatory gene programs in the superficial cortical layers, closely linked to Aβ-related processes; they validated transcriptional changes in plaque-proximal areas using 5xFAD mice [35]. Recent spatial omics studies, integrating Visium with immunohistochemistry, have directly resolved plaque–glia ‘micro-niches’ in the AD human cortex. Avey et al. (2025) [36] analyzed tissue from the posterior cingulate cortex of 21 AD patients, categorizing spatial sequencing spots based on plaque and glial markers into classes such as low-Aβ/high-gliosis. Results showed that ‘low-Aβ’ regions had more severe neuronal loss, while ‘high-gliosis’ regions displayed upregulated inflammatory pathways. Gene modules characteristic of DAM, DAA, and PIGs (plaque-induced genes) were enriched in plaque-proximal areas [36].

Furthermore, iPSC-derived multicellular culture experiments confirmed that upon Aβ exposure, microglia, but not astrocytes, adopted a reactive state consistent with the spatial transcriptomic data from human brains. Recent research has also identified a terminally inflammatory microglia (TIM) population that accumulates in aged brains and APOE4-genotype AD brains. TIMs co-express inflammatory factors (e.g., S100A8/A9) and cellular stress markers (e.g., Fos, Jun) and exhibit metabolic dysregulation and reduced Aβ clearance capacity, resembling a T-cell exhaustion-like state [37]. Studies on human AD brain tissue further corroborate the clinical relevance of microglial heterogeneity, with distinct subtypes correlating with disease stage and pathological severity [38]. Key findings from human studies are summarized in Table 3.

### 3.3. Comparison Across Disease Stages

Multiple studies revealed differences in cellular states across various stages of AD. AD progression is generally categorized into preclinical, mild cognitive impairment (MCI), and confirmed AD dementia stages. Mathys et al., in their single-nucleus analysis of the prefrontal cortex from 48 individuals, observed that microglial and astrocyte activation, along with altered neuronal expression, accompany early AD pathology [1]. Srinivasan et al. reported a progressive activation state of microglial populations that correlates with plaque accumulation in transgenic AD mice. A study based on peripheral blood transcriptomics provides new insights for ultra-early AD diagnosis. This research found that in the subjective cognitive decline (SCD) stage—the ultra-early phase preceding clinical AD symptoms—the transcriptome of peripheral blood already exhibits significant downregulation of the type I interferon (IFN-I) signaling pathway, suggesting that dysregulation of peripheral immune IFN responses may occur prior to the accumulation of typical brain pathology [40].

**Figure 3:**
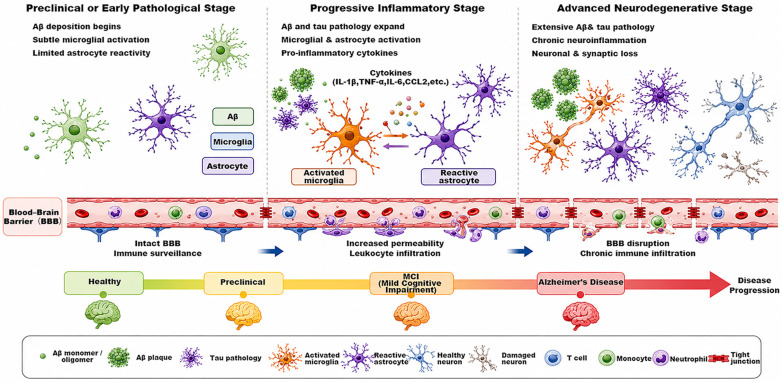
Dynamic progression of neuroinflammation and BBB dysfunction during AD pathogenesis.

Overall, the early stage of initial Aβ accumulations is characterized by microglia-mediated clearance responses and neuronal stress pathways; mid-to-late stages feature severe chronic inflammation, complement activation, and synaptic loss (Figure 3). These studies highlight the dynamic evolution of inflammatory responses with disease progression. However, due to the challenges of obtaining longitudinal time-series data from human samples, current understanding is largely inferred from cross-sectional analyses. This highlights the need for future longitudinal studies or well-designed animal models to clarify the cellular and molecular trajectory across different stages.

However, it is critical to emphasize that human AD progression is biologically heterogeneous. Individuals exhibit marked variability in age of onset, rate of cognitive decline, and co-pathologies (e.g., vascular lesions, TDP-43 pathology). The staging framework presented in Figure 3 represents a simplified model based on population-level trends and may not reflect individual patient trajectories. Furthermore, most human transcriptomic studies are cross-sectional, analyzing postmortem tissue from different individuals at different disease stages. This design infers longitudinal trajectories indirectly rather than tracking disease evolution within the same individual. Integrating postmortem transcriptomic data with longitudinal clinical and imaging data from prospective cohort studies represents a promising strategy to address this limitation.

Schematic overview illustrating the temporal progression of AD from early preclinical stages to advanced neurodegeneration. In the early pathological stage, low levels of Aβ accumulation are associated with subtle microglial activation and limited astrocyte reactivity while the BBB remains largely intact. During the progressive inflammatory stage, increasing Aβ and tau pathology promote activation of microglia and reactive astrocytes, leading to elevated cytokine production, oxidative stress, and enhanced neuroimmune signaling. BBB permeability progressively increases, facilitating leukocyte infiltration and amplification of local inflammatory responses. In advanced AD, extensive plaque and tau pathology are accompanied by chronic neuroinflammation, sustained glial activation, synaptic dysfunction, neuronal degeneration, and severe BBB disruption. Peripheral immune-cell recruitment further exacerbates tissue injury and neurodegenerative progression. The lower timeline summarizes the transition from healthy brain aging through preclinical disease and MCI to symptomatic AD, highlighting the progressive interplay among protein aggregation, neuroimmune dysregulation, vascular dysfunction, and neuronal loss. This staging scheme represents a simplified model based on population-level trends and may not reflect individual patient trajectories.

### 3.4. Spatial Transcriptomics Reveals Differences Between Aβ Plaque-Proximal and Distal Regions

Spatial transcriptomics provides direct evidence for investigating the plaque microenvironment. Studies have identified unique cellular subtypes and molecular signatures in the vicinity of Aβ plaques (proximal region), whereas tissue further away from plaques (distal region) retains a more homeostatic state. In the complex pathological course of AD, inflammation does not occur uniformly but exhibits significant spatiotemporal heterogeneity [32]. For instance, proximal regions are enriched with pathologically activated microglia which producing DAM markers like *Cst7*, *Itgax*, and *Apoe*, and reactive astrocytes which upregulate DAA markers such as *Gfap* and *Serpina3n*; these cells express complement factors and pro-inflammatory mediators, likely participating in altered cell–cell communication and synaptic signaling disruption [32,36].

Spatial in situ analysis further confirms that DAMs aggregate significantly around Aβ plaques, with proximal areas (<50 μm) showing prominent enrichment of genes related to lysosomal degradation, complement pathways, and Aβ metabolism [41]. Conversely, distal regions are dominated by homeostatic neurons and supportive cells, exhibiting lower expression of inflammatory genes. In the study by Avey et al., ‘low-Aβ/high-gliosis’ regions (representing diffuse plaques) showed significant upregulation of neuronal apoptosis markers, whereas both low- and high-Aβ regions with high gliosis were enriched for inflammation-related pathways, with a more pronounced effect observed in low-Aβ (diffuse plaque) areas [36]. Mallach et al. also reported the presence of DAM characterized by upregulated Apoe, Lpl, and Trem2, along with dysregulated astrocytic signaling around plaques, including downregulation of the GABA transporter Slc6a11 (Gat3) in astrocytes within microglia-dense plaque niches [32]. Spatial transcriptomic analysis further demonstrates a ‘core–shell’ cellular organization pattern around Aβ plaques in the AD hippocampus: microglia aggregate near the plaque core, while astrocytes are distributed more peripherally. Enhanced ligand–receptor interactions, such as SPP1-ITGB1 and MIF-CD44, between these two cell types drive local neuroinflammatory responses [33].

These findings support the ‘plaque–glia niche’ hypothesis in AD: the microenvironment created by Aβ plaques recruits and reprograms glial cells, forming localized inflammatory foci that concurrently contribute to neuronal degeneration in more distant regions (Table 4).

### 3.5. Intercellular Interaction Networks and Key Regulatory Factors

The formation and maintenance of this inflammatory microenvironment depend on a complex network of interactions among different cell types. Cell–cell communication analyses based on single-cell or spatial transcriptomic data confirm that microglia, astrocytes, and neurons are central nodes in this network [42]. In AD models, Aβ-activated microglia release signals like IL-1α, TNF, and C1q, which collectively induce astrocytes to transition towards a neurotoxic phenotype [31]. Activated astrocytes can induce neuronal and oligodendrocyte death through the release of saturated long-chain lipids, specifically free fatty acids and very-long-chain phosphatidylcholines, carried within ApoE/ApoJ lipid particles. This process is mediated via lipoapoptosis pathways, which involve FOXO3a dephosphorylation and PUMA upregulation, and occurs independently of physical cell contact. Notably, the toxic activity is mediated by saturated lipids trafficked within ApoE/ApoJ lipoparticles, rather than by the protein components themselves [43]. Concurrently, stressed neurons alter their network activity by exhibiting excitotoxicity and engaging compensatory remodeling of inhibitory inputs, thereby further influencing the functional states of microglia and astrocytes. Complex feedback and feedforward interactions exist between glial cells and neurons. This multicellular ‘cellular phase’ response can transition from an initially compensatory and protective reaction to a chronic, irreversible pathological process as the disease progresses, collectively exacerbating synaptic dysfunction and neurodegeneration [44].

Furthermore, the APOE genotype exerts pleiotropic effects on AD pathological progression. Different APOE isoforms (ε2, ε3, ε4) are closely associated with variations in Aβ plaque burden, tau pathology severity, microglial and astrocyte reactivity, and blood–brain barrier integrity, highlighting the critical role of APOE in modulating intercellular communication and disease advancement [45].

Additional studies have revealed that in the presence of amyloid plaques plaque-associated microglia can transform into neurodegenerative microglia (MGnD). These cells significantly accelerate the propagation of tau pathology across brain regions by hypersecreting extracellular vesicles carrying phosphorylated tau protein. This suggests that microglia-mediated vesicle secretion is a crucial mechanism linking Aβ pathology to the spread of tau pathology [46].

Taken together, the findings summarized above illustrate the distinct yet complementary roles of multi-dimensional transcriptomics and conventional experimental approaches. scRNA-seq and spatial transcriptomics excel at hypothesis generation—proposing frameworks of cellular microenvironments, identifying novel cell states (e.g., DAM, DAA, TIM, DAO), and providing candidate key molecules for further study (e.g., *TREM2*, *C3*, *APOE*). In contrast, conventional approaches, including knock-out mouse models, pharmacological interventions, immunohistochemistry, and functional assays in iPSC-derived cultures, are essential for causal validation and mechanistic dissection. For example, the identification of the DAM subpopulation was a direct scRNA-seq discovery, while the functional role of TREM2 in AD pathogenesis was rigorously validated through *Trem2*-deficient mouse studies and GWAS. This complementary framework is emphasized throughout the present review.

### 3.6. Cross-Study Comparison and Critical Evaluation

Direct comparison of findings across different studies reveals both robust transcriptional signatures and platform- or model-specific discrepancies. Core signatures of DAM, including upregulation of *Apoe*, *Trem2*, *Cst7*, and downregulation of homeostatic markers *P2ry12* and *Tmem119*, have been consistently replicated across multiple scRNA-seq datasets from both 5xFAD mice [27] and human AD brains [1,19], suggesting cross-species and cross-platform robustness. However, the degree of astrocyte heterogeneity reported varies considerably. Habib et al. (2020) identified a distinct DAA population in 5xFAD mice [30], whereas human snRNA-seq studies have described more continuous transcriptional gradients rather than discrete subtypes [1]. These discrepancies may reflect true species differences, model limitations (e.g., absence of robust tau pathology in 5xFAD mice), or technical factors such as dissociation artifacts and sequencing depth.

Platform-specific biases also merit consideration. MERFISH achieves near-single-cell resolution and high detection efficiency for targeted genes but is limited by panel size, whereas Visium offers whole-transcriptome coverage at a lower spatial resolution (55 μm spots). Slide-seq and Stereo-seq provide higher resolution but require fresh-frozen tissue, limiting compatibility with archived clinical specimens. These technical differences influence biological conclusions; for instance, the identification of rare cell states (e.g., TIM cells) may be more feasible with high-throughput droplet-based methods [39], while spatial-niche analysis (e.g., plaque core–shell organization) requires high-resolution imaging-based platforms [33]. A key unresolved question is which transcriptomic signatures are consistently reproducible across diverse human cohorts, platforms, and analytical pipelines, emphasizing the need for standardized benchmarking frameworks and multi-center validation studies.

## 4. Technical Limitations and Improvement Needs

Although scRNA-seq and spatial transcriptomics technologies have significantly advanced AD research, several limitations persist.

Regarding sample preparation, scRNA-seq requires tissue dissociation to obtain single cells, which can cause cellular damage and bias. For instance, some fragile neurons are easily lost or under-represented during this process, and non-neuronal cells may be over-represented in the final cell suspension. For brain tissue, snRNA-seq is often preferred to mitigate dissociation artifacts, as the nuclei are more resilient to mechanical stress and can be isolated from archived frozen specimens. Spatial technologies frequently require fresh-frozen samples, which can be challenging to obtain for clinical specimens. Notably, comparative studies of the mouse visual cortex suggest that while snRNA-seq detects fewer total transcripts per sample than scRNA-seq, the inclusion of intronic reads enables the identification of closely related neuronal cell types with a resolution comparable to whole-cell analysis [47]. This supports the utility of snRNA-seq for large-scale surveys of cellular diversity. Improvement strategies include developing spatial platforms compatible with FFPE tissues (e.g., 10x Visium FFPE kit) or optimizing frozen section pre-treatment and promoting techniques like chemical fixation or cryopreservation of nuclei in single-cell sequencing to reduce bias and batch effects.

Beyond the limitations of sample preparation and spatial resolution, it is important to recognize that transcriptomics-based approaches inherently cannot measure certain molecular classes critical to AD pathogenesis. First, extracellular vesicles (EVs) carry diverse functional molecules including mRNAs, miRNAs, and proteins, and have been implicated in the spread of tau pathology [46]. However, current transcriptomic workflows are not optimized for EV capture, as EVs are typically lost during standard RNA extraction or are present at concentrations below detection limits. Future integration of EV isolation with single-vesicle sequencing technologies is needed to bridge this gap. Second, carbohydrate-conjugated molecules, particularly gangliosides such as GM1, play critical roles in AD pathology. GM1 binds to Aβ and promotes amyloid fibril formation, yet these molecules lie beyond the detection scope of transcriptomics. Complementary lipidomic and glycomic analyses are therefore essential for a comprehensive understanding of AD pathogenesis.

Regarding spatial resolution, most sequencing-based spatial methods (e.g., Visium, Slide-seq) have a resolution limited to the multicellular/cell-population level, making it difficult to capture single-cell or subcellular differences. While imaging-based methods offer high resolution, they are often restricted by the number of probes that can be used. Improvement strategies involve combining multiple spatial technologies—for example, using MERFISH or seqFISH for deep targeted probing on regions or gene clusters of interest identified by whole-transcriptome Visium—further increasing capture spot density through technologies like Stereo-seq, or developing nanoscale spatial barcoding technologies.

In terms of throughput and coverage, high-resolution imaging methods are time-consuming and costly, limiting their application to large tissue areas or whole-brain scales [16,20]. To enhance throughput, potential strategies include incorporating new technologies like automated super-resolution microscopy and optical synthesis to increase throughput or employing clustering/partitioning strategies to pre-select regions of interest for high-resolution analysis.

The analysis of multi-dimensional transcriptomic data presents unique computational challenges. Batch effects represent a major confounder, as differences in sample processing, sequencing platforms, and reagent lots can obscure biological signals. While batch integration algorithms such as Harmony, Seurat (CCA/RPCA), and scVI have been developed to mitigate these effects, their comparative performance varies across datasets, and no single method is universally optimal [21]. For spatial transcriptomics, cell-type deconvolution—inferring the cellular composition of each capture spot from reference single-cell data—remains challenging due to technical noise, incomplete reference atlases, and spatial heterogeneity. Ligand–receptor interaction inference tools (e.g., CellChat, NicheNet) are widely used to predict cell–cell communication but suffer from high false-positive rates, necessitating orthogonal validation through experimental approaches such as knock-out models or spatial protein imaging. Finally, reproducibility across datasets remains a concern, underscoring the need for standardized analytical workflows, open-access data sharing, and multi-center validation studies.

Regarding data analysis and integration, single-cell data contain high noise and dropout events characterized by zero-inflation, while spatial data face challenges like background noise and barcode bleeding. Cell type identification often relies on marker genes, which can introduce bias across different studies [48]. Direct comparison and integration of scRNA-seq and spatial data remain challenging, necessitating the development of cross-modal alignment algorithms. Improvement directions include developing robust denoising and pseudotime inference algorithms, utilizing data fusion tools such as Seurat integration, Tangram, and LIGER to align single-cell and spatial data for inferring the spatial distribution of single-cell subtypes, and incorporating multi-omics joint analyses by leveraging proteomic or methylomic information to aid cell identification [49].

In terms of multi-omics integration, measuring only the transcriptome limits insight into regulatory mechanisms. Improvement directions include combining single-cell ATAC-seq to explore chromatin accessibility, employing CITE-seq/CyTOF to obtain protein expression data, introducing single-cell metabolomics to examine metabolic changes, developing spatial proteomics technologies such as in situ mass spectrometry imaging to pair with transcriptomics, and exploring spatial multi-omics—for example, simultaneous in situ detection of mRNA and proteins, or spatial ATAC-seq. The major limitations of the technology and improvement strategies are summarized in Table 5.

## 5. Clinical Translation Prospects

The application of single-cell and spatial omics in the AD field holds broad promise but also faces considerable challenges.

For early diagnosis, this study suggests that molecular subtypes (including those with neuroinflammatory signatures) are detectable at the MCI stage using brain transcriptomics, suggesting that pathological divergence occurs before advanced clinical decline. While this study was limited to postmortem tissue, it highlights the potential for future multi-omic and peripheral biomarker studies to translate these subtype-specific network drivers (e.g., LRP10, MSN) into clinical tools [50]. Advances in biomarker development provide tools for this goal: novel PET tracers such as those targeting TSPO, MAO-B, or the P2X_7_ receptor enable in vivo visualization of microglial activation [51]; the clinically validated blood-based biomarker GFAP offers significant potential for non-invasive clinical application in detecting Aβ pathology across the AD continuum [52]. For example, specific gene expression signatures from microglial or astrocyte subtypes might correspond to detectable circulating factors. However, multi-omic sequencing is currently too costly and technically demanding for routine clinical screening; its primary value lies in target and postmortem-derived candidate biomarker discovery, with downstream clinical application relying on lower-cost detection methods, e.g., ELISA or targeted proteomics.

Recently, plasma biomarkers identified through spatial transcriptomics screening have shown considerable promise. For instance, levels of CCK and PMP2 proteins carried within brain-derived extracellular vesicles are significantly decreased in the plasma of AD patients. Combined detection of these two markers can effectively differentiate AD from healthy controls and non-AD dementias, offering a new avenue for non-invasive diagnosis [33]. Plasma GFAP, when combined with plasma Aβ42/Aβ40 ratios, improves the discrimination of preclinical brain Aβ pathology (as defined by PET imaging) in cognitively normal older adults [53]. The integration of machine learning algorithms with multi-omics data further enhances the performance of blood-based biomarkers for early AD detection [54]. However, the high cost of omics sequencing precludes its use for large-scale sample screening; current applications primarily rely on downstream validation using lower-cost detection methods for discovered targets. Moreover, the term ‘precision medicine’ in the context of AD transcriptomics remains a potential future application rather than a current reality, given barriers in sample acquisition, cost, and the lack of longitudinal omics data at the individual patient level.

Regarding drug target identification, key pathways revealed by scRNA-seq (e.g., TREM2-dependent DAM signatures, NLRP3 inflammasome) provide targets for novel drug development. TREM2, as a crucial regulator of microglial function, is a focus of research, with genetic evidence strongly supporting agonistic approaches over antagonism [55]. Studies indicate that TREM2 agonists can enhance microglial phagocytosis and promote their transition towards a neuroprotective state, reducing neuritic dystrophy and modifying Aβ plaque morphology toward a less toxic conformation in animal models [56]. Furthermore, modulating glial activation states, such as through deletion of Chi3l1, has been shown to alter microglial functional phenotypes (e.g., enhanced CD68 expression and Aβ phagocytosis), suggesting that regulation of glial activity may mitigate amyloid pathology. However, this study relies on genetic deletion of a secreted glycoprotein rather than direct intervention in microglial transcriptional programs or metabolic states [57]. Spatial analysis of AD mouse models reveals that the complement receptor C3aR is selectively upregulated in microglia surrounding amyloid plaques, while its ligand C3 is predominantly expressed in astrocytes. This region-specific astrocyte–microglia crosstalk impairs Aβ clearance and exacerbates pathology. Pharmacological blockade of C3aR reduces plaque burden, demonstrating that targeting region-specific glial communication can disrupt neuroinflammatory cycles [58]. Complement pathway inhibitors have shown synapse-protective effects in preclinical studies, potentially representing a novel therapeutic strategy for AD [59].

For efficacy assessment and precision medicine, single-cell omics can be used to monitor treatment efficacy at the cellular level by comparing transcriptional changes in specific cell subtypes before and after drug administration. Moreover, these technologies can help identify patient subgroups, enabling precision medicine—as patients may exhibit heterogeneity in inflammatory pathways, warranting tailored treatments. However, current limitations in sample acquisition, especially biopsies, and data volume hinder widespread clinical implementation.

Overall, scRNA-seq and spatial omics offer new avenues for AD early screening and targeted therapy, but overcoming challenges related to cost, reproducibility, and data interpretation is essential for clinical translation.

## 6. Challenges and Future Directions

Despite the immense promise, targeting neuroinflammation faces numerous challenges. First, the causal relationship between specific cellular subtypes and AD pathogenesis remains unclear, and discrepancies between animal models and human disease limit clinical translation [60].

Second, selecting the optimal therapeutic time window is critical, as intervention too early or too late may compromise efficacy [5]. The dual nature of neuroinflammation regulation is also a key consideration; excessive suppression could impair its normal physiological functions [61]. Future research should focus on integrating single-cell multi-omics data (e.g., epigenomics, proteomics) and rigorously validating targets in human pluripotent stem cell-derived models and human tissue samples [62].

A recent clinical study utilizing PET imaging and fluid biomarkers further underscores the priority of targeting neuroinflammation. Consistent with multi-omics findings in postmortem tissue, this study provides in vivo confirmation in humans that microglial activation modulates astrocyte reactivity. This glial crosstalk acts as a key upstream event that triggers the subsequent propagation of tau pathology and cognitive decline in the presence of pre-existing Aβ pathology [63]. Additionally, this study establishes a causal link demonstrating that microglia-mediated T-cell infiltration drives neurodegeneration in tauopathy. It suggests that targeting the detrimental crosstalk between activated microglia and infiltrating T cells may represent a potential therapeutic strategy for AD and primary tauopathies [64]. The establishment of human tissue banks and organoid models will facilitate the transition from basic research to clinical application [65]. International collaboration and large-scale data sharing are essential for deciphering the complex mechanisms of neuroinflammation in AD [66].

To move beyond correlation toward causation, future research must prioritize orthogonal experimental validation of transcriptomic findings. This includes: (1) genetic perturbation experiments (e.g., conditional knock-out or overexpression of candidate genes such as *TREM2*, *APOE*, or *C3* in AD mouse models) to assess causal effects on pathology and cognition; (2) longitudinal in vivo imaging combined with spatial transcriptomics to track the temporal evolution of plaque-associated niches; (3) functional assays in human iPSC-derived multicellular co-culture systems (neurons, microglia, astrocytes) to test the sufficiency of identified molecular pathways in driving neuroinflammation and synaptic loss; and (4) time-resolved transcriptomic profiling of AD mouse models across the lifespan to capture the dynamic transitions between homeostatic, reactive, and exhausted glial states. These approaches will help establish whether transcriptomically defined cell states are drivers or merely consequences of AD pathology.

## 7. Conclusions and Outlook

Neuroinflammation does not act in isolation but forms a complex pathological network with other AD hallmarks. First, a vicious cycle exists between neuroinflammation and Aβ pathology: microglial dysfunction impairs Aβ clearance, while inflammatory signals promote APP processing to generate more Aβ. Second, neuroinflammation interacts with tau pathology: inflammatory factors can activate kinases, leading to tau hyperphosphorylation and promoting microglia-mediated prion-like spreading of tau. Third, neuroinflammation contributes to synaptic dysfunction and neuronal death through complement pathway-mediated aberrant synaptic pruning and direct neurotoxicity of inflammatory factors. Furthermore, vascular pathology, metabolic dysregulation, and aging-related immune changes (e.g., immunosenescence) intertwine with glial activation, collectively driving disease progression.

Neuroinflammation acts as an important ‘amplifier’ and integrator in AD pathology. Single-cell transcriptomics and spatial omics technologies are providing unprecedented insights into neuroinflammation within the AD brain. From characterizing heterogeneous microglial and astrocyte states to deciphering complex cellular interactions within the plaque microenvironment, these technologies continuously generate novel biological hypotheses and mechanistic frameworks. Targeting neuroinflammation, particularly through precise, cell-type-specific, and spatiotemporally controlled modulation, represents a crucial path forward to overcome the current therapeutic impasse in AD.

Therefore, several specific unresolved questions should be identified: (1) What are the causal roles of individual glial states (DAM, DAA, TIM, DAO) in AD progression? (2) How do plaque-associated inflammatory niches evolve over time within the same individual? (3) What mechanisms underlie the regional heterogeneity of neuroinflammatory responses? (4) How do peripheral immune cells and blood–brain barrier dysfunction contribute to brain inflammation in AD? (5) Which transcriptomic signatures are robustly reproducible across diverse human cohorts? Accordingly, several concrete future directions should be taken into account: (1) building longitudinal multi-omics datasets from prospective cohort studies combining imaging, fluid biomarkers, and eventual postmortem tissue to capture dynamic pathological changes; (2) integrating transcriptomics with proteomics, epigenomics, and metabolomics for multi-layered mechanistic insight to reveal cross-talk between molecular layers; (3) functional validation of candidate regulatory pathways using CRISPR-based perturbations in iPSC-derived glial models and organoids to establish causality; (4) systematic validation of plaque-associated inflammatory niches across different brain regions and disease stages to understand spatial and temporal heterogeneity.

Targeting neuroinflammation, particularly through precise, cell-type-specific, and spatiotemporally controlled modulation, represents a crucial path forward to overcome the current therapeutic impasse in AD. Future efforts will increasingly emphasize integrative multi-omics analyses of large-scale human cohorts and longitudinal tracking of early pathological changes. Simultaneously, advancements in algorithms and experimental techniques—such as improving sensitivity, enabling parallel single-cell multi-omics measurements, and leveraging AI-driven analysis—will further accelerate discovery. Coupling these omics achievements with clinical research holds the potential to expedite the development of early diagnostic biomarkers and precise drug targets. Through multidisciplinary collaboration and technological innovation, the translation from mechanistic insights to clinical interventions can be realized, offering new hope for individuals affected by AD.

In conclusion, single-cell and spatial omics provide an unparalleled opportunity to dissect neuroinflammation in AD. Integrating multi-omics approaches with rigorous functional validation and clinical translation represents a critical pathway toward effective interventions for AD.

## Figures and Tables

**Table 1:** Comparison of common single-cell and spatial omics platforms and their key parameters.

Plaform/ Technology	Category	Resolution	Gene Detection Capacity	Throughput	Sample Type	Main Applications/ Advantages
10× Chromium (scRNA-snRNA)	Droplet-based (Sequencing)	Single-cell/single-nucleus (~10 μm)	Whole transcriptome (~10,000–20,000 genes)	High (up to 10^4^–10^5^ cells)	Live cells, frozen tissues	Single-cell heterogeneity analysis, high throughput
10× Visium	Spatial capture (Sequencing)	~55 μm per spot (1–10 cells per spot)	Whole transcriptome (~18,000–20,000 genes)	Moderate (~5000 spots per section)	Fresh-frozen (Visium v1), FFPE (Visium v2)	Whole-transcript spatial mapping, easy operation
Slide-seqV2	Spatial capture (Sequencing)	~10 μm per bead (near-single-cell)	Whole transcriptome	High (millions of beads)	Frozen tissues	Higher spatial resolution, captures more cells
Stereo-seq	Spatial capture (Sequencing)	0.5–3 μm per spot (single-cell and subcellular)	Whole transcriptome	Ultra-high (ultra-dense arrays)	Frozen tissues	Ultra-high resolution, enables subcellular localization
MERFISH (MERSCOPE)	In situ imaging (FISH)	Single-cell /subcellular	Scalable to thousands of genes (targeted probes)	Moderate	Fixed tissues	High spatial resolution, near 100% probe capture efficiency
seqFISH+	In situ imaging (FISH)	Single-cell/ subcellular	~10,000 genes	Moderate	Fixed tissues	Ultra-high gene multiplexity, avoids optical crowding via multiple rounds of hybridization
GeoMx DSP	Region-of-interest (ROI) capture	Up to cellular level (adjustable ROI)	Targeted panels (hundreds to thousands of genes)	Low	FFPE, frozen tissues	Targeted gene panels, tissue-preserving, highly flexible ROI selection

**Table 2:** Major pathological features revealed by single-cell RNA sequencing and spatial transcriptomics in AD animal models.

Study (Year)	Sample/ Model	Technology	Species	Brain Region	Disease Stage	Key Findings	Main Inflammatory Signature	Translational Relevance	Limitations
Keren-Shaul et al. (2017) [27]	5xFAD mouse brains	scRNA-seq	Mouse	Whole brain (mainly cortex/hippocampus)	Early–mid stage (2–6 months)	Identified TREM2-dependent DAM subpopulation enriched in phagocytosis and inflammation-related genes	*Apoe*, *Trem2*, *Cst7* ↑; *P2ry12*, *Tmem119* ↓	Provides transcriptomic definition of DAM; TREM2 as therapeutic target	Early study, lacks spatial localization information
Habib et al. (2020) [30]	Hippocampus of 5xFAD mice	snRNA-seq	Mouse	Hippocampus	Early-to-late stage (2–12 months)	Identified DAA, whose abundance increases with disease progression	*Gfap*, *Serpina3n*, *Vim* ↑	Reveals astrocyte heterogeneity in AD; potential astrocyte-targeting strategies	Small sample size, mainly focused on the hippocampus
Mallach et al. (2024) [32]	Hippocampus of 5xFAD mice	Spatial transcriptomics (Visium)	Mouse	Hippocampus	Mid stage (6 months)	Abundant microglial aggregation around plaques disrupts astrocytic signaling; microglial responses are consistent across brain regions, while astrocytic responses show high regional heterogeneity	*Apoe*, *Lpl*, *Trem2* ↑ in microglia; *Slc6a11* ↓ in astrocytes	Spatial mapping of plaque–glia niche; highlights regional heterogeneity of glial responses	Mouse model only, limited to the hippocampal region
Park et al. (2023) [3]	Male App NL-G-F and 5xFAD mice/human AD brains	scRNA-seq	Mouse and Human	Cortex	Mid–late stage	Identified DAO subpopulation; elevated Erk1/2 signaling activity in DAOs, and Erk inhibition restores myelination and ameliorates AD pathology	Erk signaling ↑; myelination genes ↓	Identifies oligodendrocyte involvement; Erk inhibition as potential therapeutic strategy	Focused on oligodendrocytes, limited coverage of other cell types

Note: In the table, an up arrow (↑) indicates an upregulation, and a down arrow (↓) indicates a downregulation.

**Table 3:** Key information from selected human AD-related single-cell and spatial omics studies.

Study (Year)	Sample Type	Species	Brain Region	Disease Stage	Technology	Key Findings	Main Inflammatory Signature	Translational Relevance	Limitations
Mathys et al. (2024) [1]	283 AD and control brains (6 brain regions)	Human	Prefrontal cortex, entorhinal cortex, hippocampus, anterior cingulate cortex, etc.	MCI to severe AD (Braak stages I–VI)	snRNA-seq	Constructed an atlas of over 100 cell subpopulations; identified impaired neuronal populations in AD and linked Reelin signaling to cognitive resilience	Neuronal stress genes; glial activation signatures including MHC-II ↑ in microglia, *CLU* ↑ in astrocytes	Comprehensive reference atlas; identifies resilience-associated pathways (Reelin) for therapeutic targeting	Cross-sectional samples, no integration of spatial information
Sziraki et al. (2023) [34]	1.5 million mouse cells/ 118,000 human cells	Human and Mouse	Multiple regions (cortex, hippocampus, cerebellum)	AD and aging	scRNA-seq (EasySci)	Identified over 300 cell subtypes; revealed AD-associated transcriptional changes in rare cell types	Cell-type-specific inflammatory gene signatures	Identifies rare cell populations and their AD-associated alterations	Novel technology, requires further validation
Miyoshi et al. (2024) [35]	Brain tissues from sporadic AD (sAD) and Down syndrome-associated AD (DSAD) patients, 5xFAD mice	Human and Mouse	Cortex (superficial layers)	Early to late AD	Spatial transcriptomics + snRNA-seq	Upregulated glial inflammatory programs in superficial cortical layers; validated transcriptional changes near plaques in 5xFAD mice	*IL-1*, complement components (*C1q*, *C3*) ↑ near plaques	Reveals layer-specific glial inflammation; links Aβ to inflammatory gene programs in both sporadic and genetic AD	Differences between DSAD and sAD require further investigation
Avey et al. (2025) [36]	Posterior cingulate cortex from 21 AD patients	Human	Posterior cingulate cortex	Mild to severe AD (Braak stages III–VI)	Spatial transcriptomics (Visium) + IHC	Increased neuronal apoptosis in ‘low-Aβ’ plaque regions; upregulated inflammation in ‘high-gliosis’ regions; enrichment of DAM/DAA gene modules	DAM (*Apoe*, *Trem2*, *Cst7*) and DAA (*Gfap*, *Serpina3n*) gene modules enriched in plaque-proximal areas	Spatial mapping of plaque–glia niches in human brain; distinguishes diffuse vs. compact plaques for biomarker stratification	Relatively small number of cases
Gerrits et al. (2021) [38]	Human AD brain tissues	Human	Cortex (frontal and temporal)	Aβ and tau pathology stages	snRNA-seq	Identified microglial subpopulations specifically associated with Aβ and tau pathologies	Aβ-associated: *APOE*, *TREM2*, *CD68* ↑; Tau-associated: MHC-II genes ↑	Distinguishes Aβ-driven vs. tau-driven microglial states; potential for stage-specific targeting	Lacks spatial context information
Sun et al. (2023) [39]	Human AD brain tissues	Human	Prefrontal cortex	Aged and APOE4 AD brains	snRNA-seq	Identified terminally inflammatory microglia (TIM) subpopulation, which accumulates in aged and APOE4 AD brains and exhibits inflammatory and stress phenotypes	*S100A8*/*A9*, *Fos*, *Jun* ↑; metabolic and phagocytic genes (e.g., *TREM2*, *APOE*) ↓	Reveals exhausted-like microglial state; links aging and APOE4 genotype to microglial dysfunction; potential biomarker for late-stage AD	Mainly focused on microglia, limited analysis of other cell types

Note: In the table, an up arrow (↑) indicates an upregulation, and a down arrow (↓) indicates a downregulation.

**Table 4:** Key observations regarding Aβ plaque-proximal versus distal regions from selected studies.

Study	Sample/Model	Sample Type	Technology	Observations in Plaque-Proximal Regions	Observations in Plaque-Distal Regions
Avey et al. (2025) [36]	Posterior cingulate cortex of human AD patients	Human postmortem tissue	Visium + IHC	‘High-gliosis’ plaque regions: upregulated inflammation and AD-related pathways; enrichment of DAM/DAA gene modules	‘Low-Aβ’ plaque regions: increased neuronal apoptosis markers
Mallach et al. (2024) [32]	Hippocampus of 5xFAD mice	Mouse tissue	High-resolution spatial transcriptomics	Around plaques: dense microglial aggregation, disrupted astrocyte–neuron signaling (synaptic imbalance); elevated CD68 expression	Distal to plaques: weak glial responses, features of normal brain tissue
Miyoshi (2024) [35]	Humans and 5xFAD mice	Mouse and Human	Spatial transcriptomics + snRNA-seq	Near plaques in superficial cortical layers: upregulation of specific inflammatory genes (e.g., IL-1, complement components); transcriptomic profiles correlate with AD risk	Deep cortical layers: relatively downregulated inflammatory genes

**Table 5:** Major limitations of multi-dimensional transcriptomics and corresponding improvement strategies.

Limitation Category	Specific Issues	Current Solutions/Development Stage	Improvement Strategies
Sample Compatibility	Most spatial omics require fresh-frozen samples; scRNA-seq is limited to viable cells	Visium FFPE kit commercially available; nuclei extraction protocols well-established	Develop FFPE-compatible spatial technologies; optimize nuclei extraction and tissue fixation techniques
Spatial Resolution	Sequencing-based methods have low resolution; imaging-based methods have low throughput and limited coverage	Slide-seq (~10 μm), Stereo-seq (0.5–3 μm) available; MERFISH/seqFISH+ for targeted genes	Increase probe density; combine multiple spatial technologies (sequencing + FISH); develop high-throughput imaging protocols
Throughput and Coverage	Large-volume samples are difficult to fully cover; long imaging times	Automated microscopy platforms emerging; regional pre-selection strategies Automated microscopy platforms emerging; regional pre-selection strategies	Automated microscopy; pre-select plaque regions via regional screening; parallel sequencing of multiple fragments
Doublets and Batch Effects	scRNA-seq is prone to doublet formation; batch effects exist across different datasets	Harmony, Seurat integration, scVI widely used; doublet detection algorithms (Scrublet, DoubletFinder)	Optimize cell concentration control; apply batch integration algorithms (e.g., Harmony); implement strict quality control
Cell Type Annotation	A few cell types lack specific markers; ambiguous cell type identification	Multi-omics integration (scATAC-seq, protein markers) increasingly used; AI-assisted annotation emerging	Utilize multi-omics data (scATAC-seq, protein markers) to provide more phenotypic clues; assist annotation with AI methods
Data Integration	Direct comparison and integration of single-cell and spatial data are challenging; limited software tools	Tangram, LIGER, Seurat (Label Transfer) available but platform-specific limitations exist	Develop cross-modal alignment tools; construct comprehensive databases; share standardized analytical workflows
Reproducibility	High tissue heterogeneity makes validation across different samples difficult	Open-access data repositories (GEO, AD Knowledge Portal); multi-center consortia	Increase sample size; perform multi-center data validation; release open-access data and code

## Data Availability

No new data were created or analyzed in this study. Data sharing is not applicable to this article.

## Abbreviations

The following abbreviations are used in this manuscript:

AD	Alzheimer’s Disease
Aβ	Beta-amyloid
NFTs	Intraneuronal neurofibrillary tangles
scRNA-seq/snRNA-seq	Single-cell RNA sequencing
APP	Amyloid precursor protein
GWAS	Genome-wide association studies
BBB	Blood–brain barrier disruption
DAO	Disease-associated oligodendrocyte
FISH	Fluorescence in situ hybridization
DAM	Disease-associated microglia
DAA	Disease-associated astrocyte
DSAD	Down syndrome-associated AD
PIGs	Plaque-induced genes
TIM	Terminally inflammatory microglia
MCI	Mild cognitive impairment
SCD	Subjective cognitive decline
IFN-I	Type I interferon
MGnD	Neurodegenerative microglia
CSF	Cerebrospinal fluid
